# Impeller Configuration and Agitation Speed Govern Lignin Dispersion and Resultant Properties of Natural Rubber Composites in Wet Mixing: A CFD Experimental Study

**DOI:** 10.3390/polym18192352

**Published:** 2026-09-27

**Authors:** Zhenhua Liu, Xianzheng Liu, Yueyue Yu, Siyao Jiang, Liansong Wang, Yunfeng Qi, Huimin Lv, Lihua Jiang

**Affiliations:** 1 Department of Mechanical and Manufacturing Engineering, School of Mechanical Engineering, Shandong Huayu University of Technology, Dezhou 253034, China; 2 Department of Mechanical and Manufacturing Engineering, Faculty of Engineering and Built Environment, Universiti Kebangsaan Malaysia, Bangi 43600, Selangor, Malaysia

**Keywords:** alkali lignin, natural rubber, wet mixing, impeller configuration, computational fluid dynamics, dispersion homogeneity

## Abstract

Alkali lignin is an abundant renewable biopolymer that can both reinforce natural rubber (NR) and protect it against thermo-oxidative aging, but its strong polarity drives self-aggregation and poor dispersion in the NR matrix. Wet mixing pre-disperses lignin in the aqueous phase before compounding and is widely used to improve the dispersion of lignin relative to dry mixing; however, the initial latex–lignin mixing stage that ultimately controls the dispersion state has received little systematic attention. Here, a Eulerian–Eulerian two-fluid CFD model was established for the alkali lignin (AL)/natural rubber latex (NRL) wet-mixing system, and four impeller configurations (single- and two-stage straight-blade and 45° pitched-blade impellers) were compared at agitation speeds of 400–800 rpm. The simulations show that dispersion homogeneity is associated with the synergistic matching of macroscopic axial circulation, turbulent-fluctuation distribution, and the openness of the circulation structure. The two-stage 45° pitched-blade impeller achieved the best dispersion at all speeds, whereas the two-stage straight-blade impeller dispersed lignin worse than its single-stage counterpart because flow-region compartmentalization hindered axial particle exchange. Dispersion improved monotonically with speed, increasing rapidly from 400 to 600 rpm and showing an apparent plateau between 600 and 800 rpm. Payne effect, microstructural, vulcanization, mechanical, and thermo-oxidative aging characterizations all reproduced the dispersion ranking predicted by the model. These results clarify the role of the initial mixing stage in AL/NRL wet processing and provide a basis for impeller selection and process–parameter optimization.

## 1. Introduction

Lignin is the most abundant natural aromatic by-product from pulping, papermaking, and biomass refining industries, offering significant advantages including wide availability, low cost, biodegradability, and low density [1]. Its rigid aromatic carbon skeleton, coupled with abundant surface active groups (e.g., phenolic and aliphatic hydroxyls), endows lignin with excellent mechanical, physicochemical, and biodegradable properties, as well as antioxidant activity and thermal stability [2]. When incorporated into a natural rubber (NR) matrix as a functional filler, lignin provides mechanical reinforcement via its rigid skeleton and imparts superior anti-thermo-oxidative aging resistance through the radical scavenging effect of its intrinsic phenolic hydroxyl groups [3]. This dual functionality makes lignin an ideal green filler with great potential for high-value industrial applications [4,5].

However, the strong surface polarity of lignin promotes self-aggregation through intermolecular hydrogen bonds, forming primary agglomerates. This results in poor dispersion within the NR matrix and deteriorates the performance of rubber composites, representing the most critical challenge for lignin-filled rubber systems [6].

Current strategies to address this issue primarily involve chemical modification and processing optimization [7,8]. Chemical modification is the mainstream approach to improve filler compatibility. Techniques such as esterification, graft copolymerization, and inorganic hybridization selectively modify surface polar groups, weakening hydrogen bonding and mitigating agglomeration to enhance interfacial bonding with rubber [9]. Nevertheless, this approach has notable drawbacks. First, it often requires large amounts of organic solvents and acid/base catalysts, leading to complex procedures, high costs, and environmental pollution; second, most chemical modifications inevitably disrupt the intrinsic phenolic hydroxyl structures of lignin, compromising its natural radical scavenging capacity and causing a significant decline in the anti-aging performance of composites [10]. Balancing improved dispersion with retained antioxidant activity remains a major question.

In contrast, processing optimization preserves the intrinsic molecular structure and dual functionality of lignin, offering a cost-effective and green route to enhance dispersion [7]. Rubber mixing processes are mainly categorized into dry mixing and wet mixing [11]. Dry mixing is the most mature and widely used method in the rubber industry [12]. Researchers have attempted to improve dispersion by optimizing parameters such as mixing temperature, time, and rotor speed to enhance mechanical shear [13,14]. However, the limited shear coverage and poor uniformity of dry mixing make it difficult to completely break down dense lignin agglomerates [11]. Residual agglomerates create internal pores, interfacial defects, and stress concentration sites in the rubber matrix, failing to provide effective reinforcement and instead deteriorating mechanical strength, stability, and long-term reliability [15,16]. This technical bottleneck severely restricts the large-scale application of lignin in rubber products.

To overcome the limitations of dry mixing, wet mixing has emerged as a research hotspot in lignin/NR composite modification [17,18]. Unlike the solid-phase shear mode of dry mixing, wet mixing leverages a liquid dispersion system: lignin powder is first dispersed in water to form a stable aqueous dispersion, which is then homogeneously blended with liquid NR latex at the molecular level, followed by flocculation, drying, and refining to produce composites. This process significantly improves filler dispersion and interfacial compatibility [19,20]. Ikeda et al. [21] achieved efficient blending of sodium lignosulfonate and NR latex via a soft processing method, preparing lignin/NR nanocomposites with filler loadings of 5–40 phr. They found that lignin formed a continuous network in the NR matrix, providing strong reinforcement even at high loadings. Intapun et al. [6] systematically investigated the blending of Klason lignin with NR latex, showing that at 10 phr, lignin achieved nanoscale dispersion, increasing tensile strength and toughness by 30% and 20%, respectively, compared to pure NR. However, they noted that excessive loading re-induced agglomeration via phenolic hydroxyl hydrogen bonds, leading to performance degradation. Zheng et al. [22] combined epoxidized natural rubber (ENR) modification with wet mixing, further optimizing the interface and increasing tensile strength from 29.4 MPa to 36.2 MPa. Ferruti et al. [19] prepared masterbatches via coprecipitation of methacrylated lignin and NR latex, simultaneously improving dispersion and compatibility. Additionally, Sethulekshmi et al. [23] fabricated lignin nanoparticles (LNPs) and applied ultrasound-assisted wet mixing, achieving multi-dimensional enhancements in mechanical, antibacterial, biodegradable, and UV-shielding properties at low filler loadings.

Although wet mixing is widely used to improve the dispersion of lignin relative to dry mixing and is a core path to high-performance lignin/NR composites, existing studies predominantly focus on post-flocculation conditions, filler ratios, and solution pH, largely neglecting the regulatory role of the initial latex–lignin mixing stage. In fact, solid–liquid stirring is a prerequisite step in wet mixing [11], directly determining the suspension state of lignin particles and mixing uniformity with latex, and is crucial for preventing agglomeration. However, because conventional experimental observation cannot resolve the in-tank multi-physics fields, the correlation between flow-field structure and lignin dispersion remains unquantified, and the underlying regulatory mechanism cannot be identified. Consequently, optimization of wet mixing processes still relies on empirical trial and error.

With the advancement of computational fluid dynamics (CFD), numerical simulation has been widely applied in chemical mixing and emulsion preparation [24], becoming a powerful tool for visualizing flow, shear, and concentration fields in stirred tanks and optimizing solid–liquid mixing processes. In stirred mixing systems, impeller configuration and agitation speed are key parameters governing flow field structure, shear distribution, and solid concentration gradient [25], directly determining filler dispersion and composite quality [11]. Phumnok et al. [26] employed the Eulerian two-fluid model to investigate the flow and mixing characteristics of different Rushton turbine configurations in a silica/NR latex system, which underscores the importance of impeller geometry in the wet mixing of rubber latex.

Based on the above, this study investigates the alkali lignin (AL)/natural rubber latex (NRL) solid–liquid wet-mixing system. A Eulerian–Eulerian two-fluid CFD model is established to elucidate the flow-field-mediated regulation mechanism of lignin dispersion, with impeller configuration and agitation speed as core regulatory variables. Wet-mixing experiments under typical operating conditions are conducted to examine whether the measured performance follows the simulated dispersion ranking through Payne effect, microscopic morphology, vulcanization characteristics, mechanical properties, and anti-thermo-oxidative aging performance tests. This study aims to reveal the intrinsic mechanism of flow field regulation of lignin dispersion, fill the gap in simulation research on the initial latex–lignin mixing stage in lignin wet processing, and provide a theoretical basis for impeller selection and process parameter optimization.

## 2. Computational and Experimental Methods

### 2.1. Reactor Geometry

The geometric model for simulation was constructed based on the laboratory-scale wet mixing apparatus, and the schematic of the experimental setup is illustrated in Figure 1a. A cylindrical stirred tank with a diameter (D) of 240 mm and a liquid height (H) of 270 mm was employed for the latex-filler mixing experiments. Four equally spaced vertical baffles (thickness = 3 mm) were mounted inside the tank, and the impeller bottom clearance (L1) was set to 60 mm for all four configurations. For the two-stage configurations (DSB and D45P), the blade spacing between the two blade rows (L2) was 80 mm. The blade pitch angles of the two stages are identical, and no circumferential (azimuthal) offset is imposed between them.

Four impeller configurations were examined in this study: single-stage open six-straight-blade (SSB), single-stage open 45° six-pitched-blade (S45P), two-stage open six-straight-blade (DSB), and two-stage open 45° six-pitched-blade (D45P) impellers. The key geometric parameters of these impellers are presented in Figure 1b,c. The overall impeller diameter was 85 mm. The hub, which connects the blades, had a diameter of 25 mm and a height of 20 mm. Each blade measured 30 mm in length, 14 mm in width, and 2.5 mm in thickness, with all blades uniformly distributed at 360° in the radial direction.

### 2.2. Governing Equations

To elucidate the dispersion characteristics of alkali lignin particles during mixing with natural rubber latex, the following basic assumptions were proposed for the numerical simulation in this work:

(1) Given the low viscosity of the diluted natural rubber latex, the experimentally measured viscosity of each feed stream was adopted as a constant, and the effects of local shear rate and temperature variation on viscosity were neglected in this simplified model.

(2) The alkali lignin dispersion was treated as a liquid-like dispersed phase. Physically, the material carried by this stream is a solid lignin phase suspended in water; in the model the dispersed phase is therefore assigned the effective particle density of the alkali lignin (1.170 g·cm^−3^), together with an effective viscosity taken as the apparent viscosity measured for the as-prepared 20 wt% AL stream. The water accompanying that stream was assigned to the continuous phase and was therefore not solved as an independent component.

(3) Given that unmodified alkali lignin tends to self-aggregate via intermolecular hydrogen bonding and cannot be fully deagglomerated solely by mechanical agitation [13], the dispersed phase particles were conservatively assumed to have a single equivalent diameter of 30 μm. Since both the gravitational settling velocity and interphase slip velocity increase with particle size, a flow field capable of uniformly suspending 30 μm particles can also satisfy the dispersion requirements for smaller lignin entities.

On the basis of the above assumptions, the Eulerian–Eulerian two-fluid model was employed for the numerical simulation. In this model, the latex phase was defined as the primary continuous phase, and the alkali lignin dispersion was set as the secondary dispersed phase. Both phases were modeled as interpenetrating continuous media [26]. The conservation governing equations for each phase are expressed as follows:

(1) $$\frac{\partial (\alpha_{q}\rho_{q})}{\partial t}+\nabla \cdot (\alpha_{q}\rho_{q}u_{q})=0$$

where q is the phase index (q = l for the latex phase, the primary phase; q = s for the AL dispersed phase, the secondary phase); α, ρ and u are the volume fraction, density and velocity vector of the corresponding phase, respectively; t is time. The volume fractions of the two phases satisfy the constraint

(2) $$\alpha_{l}+\alpha_{s}=1$$

The momentum-conservation equation is

(3) $$\frac{\partial (\alpha_{q}\rho_{q}u_{q})}{\partial t}+\nabla \cdot (\alpha_{q}\rho_{q}u_{q}u_{q})=-\alpha_{q}\nabla p+\nabla \cdot \tau_{q}+\alpha_{q}\rho_{q}g+M_{q}$$

where *p* is the pressure shared by the two phases, $\tau_{q}$ is the effective stress tensor of phase q, in which the phase volume fraction has been absorbed so that the stress term in Equation (3) is written as ∇·τ_q; it comprises viscous and turbulent contributions, g is the gravitational acceleration vector, and $M_{q}$ is the interphase momentum-exchange term between the liquid and particle phases, which satisfies

(4) $$M_{l}=-M_{s}$$

Secondary interphase forces, such as virtual-mass and lift forces, are neglected; only drag and turbulent dispersion are considered. The interphase momentum exchange acting on the AL (dispersed) phase is therefore written as

(5) $$M_{s}=K_{ls}\left(u_{l}-u_{s}\right)+F_{TD,s}$$

where $K_{ls}$ is the interphase momentum-exchange (drag) coefficient between the latex and particle phases, and $F_{TD,s}$ is the turbulent-dispersion force acting on the particle phase, given by

(6) $$F_{TD,s}=-\rho_{s}D_{t,s}\frac{\nabla \alpha_{s}}{\alpha_{s}}$$

The interphase drag was described by the Schiller–Naumann model [26], for which the interphase momentum-exchange coefficient is

(7) $$K_{ls}=\frac{3\alpha_{l}\alpha_{s}\rho_{l}C_{D}\left|u_{l}-u_{s}\right|}{4d_{s}}$$

where $d_{s}$ is the equivalent diameter of the AL particles, $C_{D}$ is the drag coefficient, and $u_{l}-u_{s}$ is the relative (slip) velocity between the latex and the particle phases, whose magnitude enters the drag coefficient and the particle Reynolds number. The drag coefficient $C_{D}$ is determined by the particle Reynolds number $Re_{s}$ according to the following piecewise correlation:

(8) $$C_{D}=\left\{\begin{array}{cc} \frac{24}{Re_{s}}\left(1+0.15Re_{s}^{0.687}\right), & Re_{s}\leq 1000\\ 0.44, & Re_{s}>1000 \end{array}\right.$$

The particle Reynolds number $Re_{s}$ is calculated as

(9) $$Re_{s}=\frac{\rho_{l}d_{s}\left|u_{l}-u_{s}\right|}{\mu_{l}}$$

where $\mu_{l}$ is the dynamic viscosity of the liquid (latex) phase; the remaining symbols are defined above.

For the continuous (latex) phase, turbulence is described by the standard k–ε model, and the transport equations for the turbulent kinetic energy $k_{l}$ and its dissipation rate $\varepsilon_{l}$ are

(10) $$\frac{\partial \left(\alpha_{l}\rho_{l}k_{l}\right)}{\partial t}+\nabla \cdot \left(\alpha_{l}\rho_{l}u_{l}k_{l}\right)=\nabla \cdot \left[\alpha_{l}\left(\mu_{l}+\frac{\mu_{t,l}}{\sigma_{k}}\right)\nabla k_{l}\right]+\alpha_{l}G_{k,l}-\alpha_{l}\rho_{l}\varepsilon_{l}+S_{k,ls}$$

(11) $$\frac{\partial \left(\alpha_{l}\rho_{l}\varepsilon_{l}\right)}{\partial t}+\nabla \cdot \left(\alpha_{l}\rho_{l}u_{l}\varepsilon_{l}\right)=\nabla \cdot \left[\alpha_{l}\left(\mu_{l}+\frac{\mu_{t,l}}{\sigma_{\varepsilon }}\right)\nabla \varepsilon_{l}\right]+\alpha_{l}C_{1\varepsilon }\frac{\varepsilon_{l}}{k_{l}}G_{k,l}-\alpha_{l}C_{2\varepsilon }\rho_{l}\frac{\varepsilon_{l}^{2}}{k_{l}}+S_{\varepsilon ,ls}$$

(12) $$\mu_{t,l}={\;\rho }_{l}C_{\mu }\frac{k_{l}^{2}}{\varepsilon_{l}}$$

where $k_{l}$ and $\varepsilon_{l}$ are the turbulent kinetic energy and its dissipation rate of the liquid phase, respectively; $\mu_{l}$ is the molecular viscosity of the liquid phase, and $\mu_{t,l}$ is its turbulent (eddy) viscosity; $G_{k,l}$ is the generation of turbulent kinetic energy caused by mean velocity gradients; and $S_{k,ls}$ and $S_{\varepsilon ,ls}$ are the interphase turbulence source terms arising from the coupling between the AL dispersion and the latex. The empirical constants of the model are $C_{\mu }$ = 0.09, $C_{1\varepsilon }$ = 1.44, $C_{2\varepsilon }$ = 1.92, $\sigma_{k}$ = 1.0 and $\sigma_{\varepsilon }$ = 1.3.

### 2.3. CFD Numerical Setup

All numerical simulations were performed using the commercial software ANSYS Fluent 2022. Considering the complex multiphase flow characteristics inside the stirred tank, the multiple reference frame (MRF) method was adopted to handle the rotational motion of the impeller, and a hybrid mesh strategy was employed to discretize the computational domain. Owing to the complex geometry of the rotating domain, unstructured tetrahedral meshes were generated in this region, with local refinement applied to the impeller rotating zone to accurately capture the high-shear flow and near-wall effects. Structured hexahedral meshes were used in the stationary region and the near-wall area at the tank bottom. The tank wall, tank bottom, and baffles were treated as stationary walls with a no-slip boundary condition, whereas the impeller blades, hub, and shaft were defined as rotating walls rotating at the same speed as the rotating fluid zone.

The pressure–velocity coupling was handled by the Phase Coupled SIMPLE algorithm. To enhance solution accuracy, second-order upwind schemes were applied to the momentum, turbulent kinetic energy, and turbulent dissipation rate equations, while the volume fraction was discretized using the QUICK scheme. The standard wall functions were employed for the near-wall turbulent region.

The calculations were transient. The time step was 0.002 s, and 15000 time steps (30 s of physical time) were advanced for each configuration. The domain was initialized by a standard initialization (α_s_ = 0), after which the AL-rich feed region, corresponding to the bottom 72.5 mm layer of the tank, was patched to the AL-rich feed composition (αs = 0.10); this reproduces the experimental procedure, in which the diluted NRL is charged into the AL dispersion under continuous agitation, so that each calculation tests whether a given impeller configuration can sustain the suspension rather than create it.

The AL-rich stream served as the high-viscosity dispersed phase and the diluted NRL emulsion acted as the low-viscosity continuous phase. The physical properties of the two phases are summarized as follows: for the dispersed phase, the effective density was 1.170 g·cm^−3^, taken as the effective particle density of the alkali lignin, and the effective dynamic viscosity was 0.106 Pa·s, taken as the apparent viscosity of the as-prepared 20 wt% AL stream; for the continuous phase, the density was 0.98 g·cm^−3^, and the dynamic viscosity was 8.65 × 10^−3^ Pa·s. The water carried by the AL stream was not solved as an independent component. The AL dispersion was prepared at 20 wt% solids (water/AL = 4:1 by mass), and the NRL was diluted to ~20 wt% total solids. Referred to 100 g of dry rubber, the charge consisted of 20 g AL dispersed in 80 g deionized water and 500 g diluted latex (100 g dry rubber + 400 g water), corresponding to an overall AL volume fraction of approximately 3% over the domain.

### 2.4. Grid Independence and Convergence

The convergence criteria were set as follows: the residuals of the continuity, momentum, turbulent kinetic energy, and dissipation rate equations all fell below 10^−4^. The calculation was considered converged when the relative fluctuations of the monitored weighted volume fraction standard deviation of the AL volume fraction remained stable. To ensure computational accuracy and eliminate the influence of mesh density on the results, three mesh schemes with different densities were constructed for grid independence verification. The grid-independence study was carried out for all four impeller configurations at the highest agitation speed of 800 rpm. The weighted volume fraction standard deviation of the AL secondary-phase volume fraction was selected as the verification indicator, and the equation is as follows:

(13) $$\sigma ={\left[\frac{\sum_{i}V_{i}{\left(\alpha_{i}-ᾱ\right)}^{2}}{\sum_{i}V_{i}}\right]}^{\frac{1}{2}}$$

where $V_{i}$ is the volume of cell i, $\alpha_{i}$ is the AL volume fraction in that cell, and $ᾱ=\frac{\sum_{i}V_{i}\alpha_{i}}{\sum_{i}V_{i}}$ is the corresponding volume-averaged AL volume fraction. The quantity is dimensionless, and a smaller value corresponds to a more homogeneous dispersion.

The verification results are listed in Table 1. When the mesh number was further increased from approximately 680,000 to 960,000, the relative deviations of the weighted volume fraction standard deviation remained basically unchanged. Considering both computational accuracy and efficiency, the approximately 680,000-mesh scheme was adopted for all subsequent simulations.

### 2.5. Materials and Composite Preparation

Alkali lignin (purity 98%, D90 < 1 μm) was purchased from Beijing Huameike Biotechnology Co., Ltd. (Beijing, China). Natural rubber latex (solid content 60%) was purchased from Shandong Xinzexiang Chemical Co., Ltd. (Dongying, China). The rubber formulation was as follows: NR 100 phr, AL 20 phr, ZnO 5 phr, stearic acid 2 phr, antioxidant 4020 1 phr, accelerator NOBS 1 phr, and sulfur 2.5 phr. All other compounding additives were commercial industrial-grade products.

The processing flow is illustrated in Figure 2. First, AL particles were added to deionized water at the formulated ratio and stirred at 500 rpm for 10 min to achieve preliminary deagglomeration of AL agglomerates via mechanical shear, thereby preparing the AL aqueous dispersion. The NRL dilution with a solid content of approximately 20% was then added to the AL dispersion, followed by mechanical stirring at a set speed (400, 600, or 800 rpm) for 20 min. After stirring, a 3 wt% CaCl_2_ solution was added dropwise to the mixture under identical flocculation conditions (dropping rate, stirring speed, and temperature) to flocculate the composite, yielding the AL/NR masterbatch. The masterbatch was sliced and dried in a vacuum oven at 60 °C to constant weight.

Subsequently, the dried masterbatch was mixed with other compounding ingredients in an internal mixer at 70 rpm and an initial temperature of 90 °C. After preheating the mixing chamber, the masterbatch was added. All ingredients except the accelerator NOBS and sulfur were added 1 min after the masterbatch. The mixing continued for approximately 7 min at 120–125 °C, before dumping. After cooling to room temperature, the compound was processed on a two-roll mill with a roll gap of 1.4 mm. NOBS and sulfur were added, and the compound was passed through the mill 8 times with repeated folding (triangle-folding and tight milling) before sheeting out. The final compound was allowed to mature at room temperature for 8 h to relieve internal stress and stabilize processability. Finally, vulcanization was carried out on a plate press at 150 °C and 10 MPa. The cure time was set to 1.3 times the optimum cure time (Tc90) determined by rheometry to ensure complete formation of the cross-linked network.

### 2.6. Characterization and Testing Methods

The vulcanization properties were measured using a rotorless rheometer (M-4130C, GOTECH Technology Co., Ltd., Taizhong, China) according to ASTM D5289 [27] at 150 °C. The Payne effect was evaluated using a rubber process analyzer (RPA2000, Alpha Technologies Inc., Akron, OH, USA) via strain sweep at 60 °C. The dispersion state of AL particles was observed on tensile fracture surfaces of AL/NR composites by scanning electron microscopy (SEM, JSM-7500F, JEOL Ltd., Tokyo, Japan) after gold sputtering. Tensile properties were tested using a universal testing machine (UCAN-UT2000, UCAN Dynatex Inc., Changhua, China) at 500 mm/min according to ASTM D412 [28]. For thermal oxidation aging, dumbbell specimens were placed in a hot air aging oven according to ASTM D573 [29] and aged at 100 °C for 48 h. After aging, the specimens were conditioned in standard laboratory atmosphere before testing, and the retention rates of tensile strength and elongation at break were recorded. The tensile strength and elongation at break are reported as the arithmetic mean of four specimens, whereas the vulcanization parameters were obtained from specimens cut from the central region of the milled sheet of each compound. For the Payne effect parameters, three measurements were carried out on each compound. The G′–strain curve shown in the figures is the one whose ΔG′ value is closest to the mean of the three measurements. Additional measurements were performed only when the ΔG′ values deviated by more than 5% from their mean, to ensure acceptable repeatability. The viscosities of the diluted latex (20 wt% total solids) and the AL solution (20 wt%) were measured using a rotational viscometer (NDJ-8S, Hengping Co., Ltd., Shanghai, China) at 25 °C and 60 rpm, with spindle No. 0 for the latex and No. 2 for the AL solution. The values are reported as apparent viscosities under these conditions.

## 3. Results and Discussion

The hydrodynamic characteristics of different impeller configurations influence AL dispersion in the wet-mixing process. The regulatory effect operates through two complementary mechanisms: (1) macroscopic circulation, where axial transport keeps particles suspended and suppresses gravitational settling, whereas radial jets provide intense local shear in the impeller discharge region; (2) the spatial distribution of turbulent kinetic energy (TKE), which is used as an indicator of the intensity and spatial coverage of turbulent fluctuations. In addition, circulation efficiency near the free surface is critical for interfacial contact between AL and latex during the subsequent flocculation step. Based on this framework, this section analyzes the macroscopic velocity field, TKE spatial distribution, and solid volume fraction distribution for the four impeller types.

### 3.1. Macroscopic Flow Field Characteristics and Velocity Distribution

Figure 3 shows the velocity vector distribution on the central longitudinal section of the reactor for four impeller types at 600 rpm, clearly revealing the macroscopic flow patterns, circulation vortex structures, and spatial flow velocity distribution throughout the reactor. These flow features provide the hydrodynamic basis for analyzing the AL dispersion behavior.

Under the action of a single-stage six-blade straight turbine, a typical radial-dominant flow pattern is observed in the tank. The rotating blades drive the fluid to eject radially at high velocity along the normal direction of the blade tips. Upon impacting the tank wall, the fluid splits and flows upward and downward along the wall surface, respectively, before recirculating to the central impeller region. Two symmetrical circulation vortices are thus formed in the lower impeller region. The local radial jet intensity at the blade tips is high, resulting in pronounced local shear. However, since the high-velocity zone is mainly concentrated in a narrow annular band at the blade tips, the fluid velocity in the upper region of the impeller decays rapidly with increasing height, leading to significantly insufficient circulation driving force in most near-surface areas. For the single-stage 45° six-blade pitched turbine, the dominant flow pattern in the tank differs markedly from that of the straight-blade turbine: the blade inclination angle exerts a continuous axial pumping effect, significantly enhancing the axial fluid motion. It is worth noting, however, that although the circulation loops induced by both sides of the blades are expanded within the tank space, the flow near the liquid surface remains weak under both single-stage configurations.

Compared with the single-stage impeller arrangement, the two-stage configuration effectively improves the fluid flow state near the liquid surface. The two-stage straight-blade turbine expands the high-velocity zone into two operating planes (upper and lower), each inducing an independent circulation loop. Four relatively isolated circulation cells are formed throughout the tank, and the low-velocity stagnant zone near the liquid surface is significantly compressed, which to some extent alleviates the interface renewal bottleneck during the flocculation stage. The two-stage pitched-blade turbine further superimposes the axial pumping effects of the two blade layers, constructing a larger-scale axial dual-circulation loop within the tank, and the near-surface flow is fundamentally enhanced.

From the perspective of wet-mixing preparation of rubber composites, the large-scale vortex structure and full-domain circulation enhancement brought by the two-stage structure generally facilitate the transport of AL particles and improve the dispersion of AL fillers in NRL. Moreover, the enhanced flow intensity near the liquid surface provides hydrodynamic assurance for sufficient interfacial contact between AL and the latex phase during the flocculation stage.

### 3.2. Turbulent Kinetic Energy Distribution

Figure 4 presents the contour plots of turbulent kinetic energy (TKE) distribution on the central longitudinal section of the reactor for the four stirring configurations, visually illustrating the axial attenuation trend, the spatial extent of high-value regions, and the overall coverage morphology of TKE. Significant differences exist in both TKE intensity and spatial distribution among the different impeller types. For the single-stage six-blade straight turbine, the high-TKE zone is highly concentrated on both sides of the blade tips, forming symmetrical patches of elevated TKE. The local peak values are significantly higher than those of the pitched-blade configurations at the same level, indicating a region of high turbulent velocity fluctuation. However, the high-value zone decays rapidly along the normal direction of the blades, meaning that the region of high TKE is mainly confined to a narrow region near the impeller, with limited extension toward the liquid surface. In contrast, the single-stage 45° six-blade pitched turbine exhibits a slightly lower local TKE peak, but the axial extent of the high-TKE zone and its distribution near the liquid surface are markedly increased. The TKE attenuation in the upper region is relatively gradual, presenting a continuous and gradually diffusive characteristic.

When the impeller stage is increased to two-stage, the TKE distribution for both blade types expands from a single patch to two independent upper and lower high-value patches. For the two-stage straight-blade configuration, the two high-TKE zones are isolated from each other, with an obvious TKE valley in the transition region between the two impeller layers. The axial continuity is insufficient, failing to form a continuous turbulent action zone throughout the full height of the reactor. The two-stage pitched-blade turbine also exhibits a dual-patch structure. Due to the sustained axial pumping effect of the two blade layers, the TKE action region in the inter-layer region changes more significantly, the spacing distribution characteristics of the two high-TKE regions are more pronounced, and the action region near the liquid surface shows local attenuation.

The aforementioned differentiated TKE distribution characteristics indicate that different impeller types exhibit significant differences in the intensity and spatial continuity of turbulent fluctuations.

### 3.3. Alkali Lignin Dispersion Homogeneity

To clarify the regulatory mechanism of macroscopic flow field distribution characteristics on AL dispersion behavior in the wet-mixing system under different impeller configurations, this section employs the spatial distribution contours of solid phase volume fraction and statistical dispersion as evaluation metrics. It systematically analyzes the mixing homogeneity differences in AL fillers across the four impeller configurations and reveals the comprehensive regulatory mechanism of flow field structure and turbulence characteristics on solid dispersion.

Figure 5 presents the contour plots of AL solid phase volume fraction on the central longitudinal section at 600 rpm, which directly reflect the spatial dispersion state and concentration stratification characteristics of AL fillers in NRL. As the straight-blade panels [(c), (d)] exhibit markedly stronger local AL accumulation, the contour ranges are set separately for the pitched-blade [(a), (b)] and straight-blade panels to resolve their respective concentration gradients. For the 45° pitched-blade series, the solid phase distribution pattern is in high agreement with the aforementioned flow field and TKE characteristics. Limited by the single-stage arrangement, the single-stage pitched-blade impeller provides insufficient circulation driving force near the liquid surface, making it difficult for AL particles to be effectively transported to the upper part of the reactor. This results in a lower solid phase proportion near the surface and a larger axial concentration gradient. In contrast, the two-stage pitched-blade impeller relies on the large-scale axial dual-circulation loop formed by the superposition of two blade layers. AL particles can not only complete top-to-bottom transport throughout the reactor under the action of macroscopic circulation, but also be kept in continuous suspension, so that local re-concentration of the dispersed phase is reduced. Consequently, the spatial gradient of the solid phase volume fraction within the reactor is significantly reduced, and the dispersion effect is markedly improved, overall following the expected “two-stage over single-stage” trend.

However, the straight-blade series exhibits a trend opposite to the above pattern. Based on the previous flow field characteristics, the two-stage six-blade straight turbine indeed achieves an overall improvement in axial velocity compared to the single-stage configuration, theoretically providing better hydrodynamic conditions for AL dispersion. However, the solid phase distribution results show that local AL enrichment is more pronounced in the two-stage straight-blade system, with greater differences in solid concentration among individual circulation cells. The overall dispersion homogeneity not only fails to improve with the increase in impeller stages but is actually inferior to that of the single-stage straight-blade turbine.

To further investigate this phenomenon, a quantitative analysis was conducted on the axial average velocity distribution along the longitudinal section of the reactor and the axial velocity distribution on the mid-plane cross-section for the four configurations. The analysis reveals that although the axial average velocity and the previous macroscopic flow field both indicate that the two-stage straight-blade turbine expands the axial coverage of the high-velocity zone to a certain extent, the four circulation cells induced by the two radial jets lack effective axial communication channels, leading to a significant decrease in the axial velocity between the upper and lower circulation vortices. Once AL particles enter a certain circulation cell, they can hardly achieve sufficient migration and redistribution between the upper and lower regions. Therefore, the increase in impeller stages instead exacerbates AL deposition due to the compartmentalization of flow regions.

### 3.4. Effect of Agitation Speed and Synergistic Matching

To further investigate the effect of operating speed on dispersion, Figure 6 shows the variation in the weighted volume fraction standard deviation of AL phase volume fraction with agitation speed for the four stirring configurations.

Overall, the dispersion homogeneity of all four configurations improves monotonically with increasing speed. Throughout the entire speed range, the ranking consistently follows: two-stage 45° pitched-blade being the best, single-stage 45° pitched-blade next, single-stage straight-blade again, and two-stage straight-blade the worst. This indicates that the effect of blade inclination and stage number on the dispersion ranking is consistent across the tested speeds, reflecting the intrinsic characteristics of the flow-field structure. The observation that the two-stage straight-blade configuration performs worse than the single-stage configuration persists stably in the 400–800 rpm range, further supporting the negative impact of flow region compartmentalization induced by the two-stage straight-blade arrangement on dispersion. In contrast, the two-stage 45° pitched-blade configuration, relying on its open axial dual-circulation structure, achieves the most uniform AL distribution over the entire speed range, demonstrating a configuration-dominated dispersion advantage. Furthermore, the sensitivity of dispersion homogeneity to agitation speed differs markedly across speed intervals: in the 400–600 rpm interval, dispersion quality improves substantially, corresponding to the rapid enhancement zone dominated by axial pumping capacity; in the 600–800 rpm interval, the improvement clearly reaches a plateau with diminishing marginal returns.

Based on the comprehensive analysis of the above macroscopic flow field characteristics and AL spatial distribution results, it can be concluded that the dispersion homogeneity of AL in the latex system is not determined by a single factor such as local turbulent intensity or macroscopic circulation scale. Rather, it depends on the synergistic matching of three factors: macroscopic axial circulation capacity, turbulent-fluctuation distribution, and circulation structure openness. Although straight-blade configurations possess higher local TKE peaks, their inherent deficiency in axial transport capability prevents them from solving the problems of particle sedimentation and global diffusion. For straight-blade configurations, the two-stage arrangement further deteriorates the overall dispersion due to flow region compartmentalization. On the other hand, although pitched-blade configurations have lower local TKE peaks, their excellent axial circulation capacity and open circulation structure enable continuous transport of AL fillers throughout the entire reactor. For pitched-blade configurations, the two-stage arrangement further enhances the continuity of axial mass transfer.

This dispersion difference determined by impeller configuration serves as a crucial link connecting the processing flow field and the final material performance, which may influence the cross-linking network construction and macroscopic service behavior of the compounded rubber. The following sections will conduct systematic tests and analyses on vulcanization characteristics, dynamic viscoelasticity, and anti-thermo-oxidative aging behavior to reveal the influence mechanism of initial mixing-stage flow field regulation on the final performance of AL/NR composites.

### 3.5. Experimental Corroboration and Composite Performance

To examine whether the composite performance follows the dispersion ranking predicted by the CFD simulations, representative composite groups covering the simulated dispersion gradient were selected in this section. Four impeller configurations were used for composite preparation at the same reference rotational speed of 600 rpm (DSB-600, SSB-600, S45P-600, D45P-600), aiming to investigate the effect of impeller configuration on composite performance under identical mixing conditions. In addition, D45P samples were prepared at rotational speeds of 400 rpm and 800 rpm (D45P-400, D45P-800) to examine the influence of stirring speed on composite performance.

#### 3.5.1. Payne Effect Analysis

The dispersion state of fillers in the rubber matrix directly determines the dynamic viscoelastic behavior of unvulcanized rubber. The Payne effect is a classical indicator of filler agglomeration and dispersion homogeneity and is quantified by ΔG′, the difference between the initial storage modulus (G′) and the modulus at high strain. A smaller ΔG′ indicates more uniform filler dispersion, lower agglomerate content, and weaker destruction of the filler network under strain [30].

Figure 7a shows the variation in the storage modulus (G′) of AL/NR composites prepared with different impeller configurations as a function of strain amplitude. All samples exhibit typical Payne effect characteristics: G′ remains at a high level in the low-strain region, and as the strain increases, the filler network is gradually destroyed under shear, causing G′ to decrease monotonically until it converges at high strain. The comparison of ΔG′ in Figure 7b intuitively reflects the differences in filler network strength among the samples.

Comparing samples with different impeller types, the DSB sample exhibits the largest ΔG′ among the six groups, indicating the highest degree of AL agglomeration in this system and the most severe network destruction under strain. The ΔG′ of the SSB sample is slightly lower than that of DSB but still at a high level, suggesting that although agglomeration is alleviated, the overall dispersion state remains unsatisfactory. This is consistent with the simulation ranking where the single-stage straight-blade impeller performs better than the two-stage straight-blade in terms of dispersion. The Payne effect of the S45P sample is further weakened, while the D45P series is overall at the lowest level, with the lowest initial G′ and the most gradual decline.

Regarding the effect of agitation speed, the ΔG′ of the two-stage 45° pitched-blade system gradually decreases with increasing speed, indicating that the physical filler network continuously weakens and dispersion is further optimized. The ΔG′ decreases more significantly in the 400–600 rpm range, corresponding to the stage in which dispersion improves most rapidly at low speeds. In the 600–800 rpm range, the change in ΔG′ tends to be gentle, suggesting that further increasing the speed has limited effect on dissipating the physical network, showing diminishing marginal returns.

Comprehensively comparing the ΔG′ of all six samples, the Payne effect intensity decreases progressively with improved dispersion: the two-stage straight-blade sample is the strongest, followed by the single-stage straight-blade, then the single-stage 45° pitched-blade, and the two-stage 45° pitched-blade series is the weakest overall, with the 800 rpm sample being the best, 400 rpm relatively higher, and 600 rpm in between. This is consistent with the simulation prediction trend, supporting the regulatory mechanism of impeller type and speed on AL dispersibility.

#### 3.5.2. Microscopic Morphology

While the Payne effect reflects the overall strength of the filler network at a macroscopic statistical level, it cannot visually present the microscopic dispersion morphology of AL particles in the rubber composite. To trace the structural origin of these rheological differences and examine the flow-field regulation mechanism at the microscopic scale, the tensile fracture surfaces of the composites were examined by scanning electron microscopy (SEM), as shown in Figure 8.

At a stirring speed of 600 rpm, the fracture surface morphologies of the four samples prepared with different impeller types differ significantly. The DSB sample has a rough and uneven fracture surface, with large-sized AL agglomerates clearly visible. The interface between the agglomerates and the rubber matrix is sharp and distinct, with obvious debonding gaps and cavities left by pulled-out agglomerates. This indicates weak interfacial bonding. These observations are consistent with the agglomerate network contributing to the Payne effect. Although the agglomerate size in the SSB sample is slightly reduced compared to DSB, there are still many obvious particle aggregates, and the matrix continuity is still largely compromised. For the pitched-blade series, the agglomerate size in the S45P sample is further reduced, with only a few small-sized particle aggregates distributed, and the matrix continuity is significantly improved. The D45P sample exhibits the smoothest and most continuous fracture surface, with AL uniformly dispersed in the rubber matrix as fine particles of several microns, without obvious large-sized agglomerates. The interface between the filler and the matrix is blurred, indicating good interfacial bonding.

In addition, the fracture surfaces of the D45P series prepared at different speeds reveal an obvious staged evolution of the microscopic dispersion state with increasing speed. At 400 rpm (Figure 8e), a certain number of particle aggregates are still visible, with uneven particle distribution in some areas, and the dispersion homogeneity needs improvement. When the speed is increased to 600 rpm (Figure 8d), particle agglomeration is significantly improved, AL achieves uniform dispersion, and the flatness of the fracture surface is greatly enhanced. When further increased to 800 rpm, the fracture morphology shows no obvious difference from that at 600 rpm, with similar particle sizes and spatial distribution states.

The above microscopic morphology observations mutually corroborate the flow field simulation and Payne effect analysis results, further supporting the important role of impeller configuration and operating speed in regulating the AL dispersion state by controlling the flow field structure.

#### 3.5.3. Vulcanization Characteristics

Table 2 shows the vulcanization properties of the AL/NR composites. Under different impeller configurations and operating speeds, the vulcanization characteristics of AL/NR compounds exhibit regular evolution, and their variation trend is highly consistent with the AL dispersion homogeneity in latex. This behavior arises from the synergistic regulation of vulcanization by the filler dispersion state through both the physical filler network and the chemical cross-linking network.

From the perspective of impeller configuration effect, under the same speed condition, the vulcanization characteristics of the AL/NR composites vary progressively in the order of D45P, S45P, SSB, and DSB. The minimum torque (ML) shows a gradual decreasing trend, which is consistent with the weakening of the initial physical filler network as the AL agglomerates become more homogeneously dispersed and refined, and would be expected to correspond to improved initial flowability of the compound [31]. In contrast, the maximum torque (MH) shows a slight increasing trend. This may be attributed to the fact that the gains from improved cross-linking uniformity and interfacial reinforcement outweigh the torque loss caused by dissipation of the physical filler network [32].

The torque difference (MH−ML), which is commonly interpreted as a measure of effective cross-linking density and filler reinforcement efficiency after subtracting the initial network contribution, shows a significant increasing trend. This trend suggests that uniformly dispersed [33] AL particles are expected to contribute more effectively to the rigid skeleton role, with simultaneous improvement in the integrity of the cross-linking network and interfacial load transfer efficiency.

Both the scorch time (t10) and the optimum cure time (t90) show a gradual extension trend with improved dispersion. This behavior can be attributed to the adsorption of sulfenamide accelerators by the phenolic hydroxyl groups on the AL surface: better dispersion would be expected to yield finer particles with larger specific surface areas and more exposed phenolic hydroxyl groups, which could strengthen accelerator adsorption. This may delay the generation of vulcanization active centers and the overall cross-linking reaction process, thereby correspondingly improving scorch safety [34]. At the same time, improved dispersion is also expected to make the accelerator distribution more uniform in the matrix, which helps avoid premature cross-linking caused by locally excessive concentration, and improve the vulcanization flatness.

From the perspective of agitation speed effect, for the two-stage pitched-blade system, as the agitation speed increases, each vulcanization parameter continues the above change rule: ML gradually decreases, MH and MH−ML gradually increase, and t10 and t90 gradually extend. It is worth noting that the parameter changes show obvious nonlinear characteristics, with more significant changes in the low-speed range and a clear slowing trend in the high-speed range, which is completely consistent with the speed response law of dispersion.

#### 3.5.4. Mechanical Properties

Figure 9 shows the tensile strength and elongation at break of AL/NR vulcanizates under different impeller configurations and operating speeds, respectively. Overall, the evolution of mechanical properties is highly consistent with the aforementioned improvement in filler dispersion homogeneity.

From the perspective of impeller configuration effect, under the same speed (600 rpm) condition, both tensile strength and elongation at break decrease progressively in the order of D45P, S45P, SSB, and DSB. The DSB-600 sample with the worst dispersion has the lowest values for both indicators (17.8 MPa and 584.0%, respectively), while the D45P-600 sample with the best dispersion reaches the highest (21.8 MPa and 643.0%, respectively). This indicates that the uniform dispersion of AL effectively reduces interfacial defects and enhances the load transfer efficiency between the filler and the matrix.

From the perspective of agitation speed effect, for the two-stage 45° pitched-blade system, both mechanical indicators show a significant jump when the speed increases from 400 rpm to 600 rpm. When the speed further increases from 600 rpm to 800 rpm, the indicators only show a slight increase (tensile strength from 21.8 to 21.9 MPa, elongation at break from 643% to 652%). This further indicates that after the speed reaches a certain level, the AL dispersion shows limited further improvement under this configuration, and further increasing the speed has limited marginal benefit for optimizing mechanical properties.

#### 3.5.5. Anti-Thermo-Oxidative Aging Performance

The key advantage of alkali lignin over traditional inorganic fillers is that its phenolic hydroxyl groups can scavenge free radicals, endowing the rubber composites with intrinsic thermo-oxidative aging resistance [2,3]. The effective exposure degree of phenolic hydroxyl active sites and the interfacial bonding state are expected to depend on the filler dispersion homogeneity [7].

The thermo-oxidative aging test results are presented in Figure 10, which indicate that the differences in performance retention rates of each sample have a good correspondence with AL dispersion homogeneity revealed in the previous text. The variation law is intrinsically consistent with the regulatory mechanisms of vulcanization characteristics and mechanical properties. After thermo-oxidative aging, the tensile strength and elongation at break of all samples decrease to varying degrees, which is the result of the combined effects of oxidative degradation of rubber molecular chains and evolution of the cross-linking structure under heat [35]. However, the degree of performance deterioration under different dispersion states varies significantly.

From the perspective of impeller configuration effect, under the same speed condition, both the AL dispersion and the retention rates of tensile strength and elongation at break vary progressively in the order of D45P, S45P, SSB, and DSB. In the two-stage straight-blade system with the worst dispersion, alkali lignin mostly exists as large-sized agglomerates: on the one hand, the phenolic hydroxyl groups inside the agglomerates are expected to be less accessible to the peroxy and alkoxy radicals generated during thermo-oxidative aging, so that the utilization of the active sites would be reduced and the aging chain reaction would be less effectively interrupted [36]; on the other hand, the interfacial defects and pores between the agglomerates and the matrix may provide preferential channels for the inward diffusion of oxygen and heat, thereby promoting the oxidative degradation inside the matrix [31]. These two effects are considered to account for the relatively poor anti-aging performance. With improved dispersion, the AL particles are refined and more uniformly distributed in the matrix, which would increase the exposure of the phenolic hydroxyl active sites and reduce the interfacial defects. The anti-thermo-oxidative aging performance is therefore expected to strengthen progressively as the dispersion improves.

From the perspective of agitation speed effect, for the two-stage 45° pitched-blade system, when the speed increases from 400 rpm to 600 rpm, both performance retention rates are significantly improved, corresponding to the optimization process from insufficient dispersion at low speed to sufficient dispersion. Combined with the speed response law of dispersion revealed earlier, it can be inferred that when the speed is further increased, the improvement amplitude of anti-aging performance will gradually narrow, showing the characteristic of diminishing marginal return, which is consistent with the nonlinear variation trend of vulcanization and mechanical properties.

## 4. Conclusions

In this study, a Eulerian–Eulerian two-fluid CFD model was established to investigate the dispersion behavior of alkali lignin (AL) in natural rubber latex (NRL) under wet mixing, with the impeller configuration (single-stage straight-blade, single-stage 45° pitched-blade, two-stage straight-blade, and two-stage 45° pitched-blade) and agitation speed (400–800 rpm) as the core variables. The simulations revealed that the dispersion homogeneity of AL was associated with the synergistic matching of macroscopic axial circulation capacity, turbulent-fluctuation distribution, and circulation structure openness. Among the four configurations, the two-stage 45° pitched-blade impeller achieved the most uniform AL dispersion across the entire speed range, owing to its open axial dual-circulation structure. In contrast, the two-stage straight-blade impeller resulted in poorer dispersion than its single-stage counterpart, because the two radial blade sets compartmentalized the flow field and lacked axial connectivity. Dispersion homogeneity improved monotonically with increasing speed for all configurations, with more pronounced enhancement in the 400–600 rpm range and an apparent plateau between 600 and 800 rpm.

Experimental characterization was carried out on representative groups. The Payne effect analysis showed that the storage modulus difference (ΔG′) decreased progressively from DSB-600 to D45P-600, indicating reduced filler network strength. SEM observations indicated that the D45P-600 sample exhibited the finest and most uniform AL particle distribution with minimal agglomerates. Vulcanization tests revealed that improved dispersion led to a gradual decrease in minimum torque and increases in torque difference, scorch time, and optimum cure time. Mechanical testing showed that tensile strength and elongation at break increased from 17.8 MPa and 584.0% for DSB-600 to 21.8 MPa and 643.0% for D45P-600. Thermo-oxidative aging tests demonstrated that the retention rates of tensile strength and elongation at break improved from 71% and 67.7% for DSB-600 to 82.2% and 76.8% for D45P-600. These experimental results were consistent with the dispersion trends predicted by the CFD simulations, supporting the regulatory role of impeller configuration and agitation speed on AL dispersion and its subsequent impact on composite performance.

It should be noted that the present study has certain limitations. Geometrically, only the current laboratory-scale configuration was examined, whereas engineering applications would additionally need to account for impeller arrangement, baffle structure, and temperature. With respect to the material system, the physicochemical properties of different lignins—and the rheological differences they introduce—may influence simulation accuracy and therefore warrant further investigation. Finally, coupling a population balance model with dedicated experimental measurements to elucidate the relationship between impeller geometry and agglomerate size offers a promising direction for future work.

## Figures and Tables

**Figure 1:**
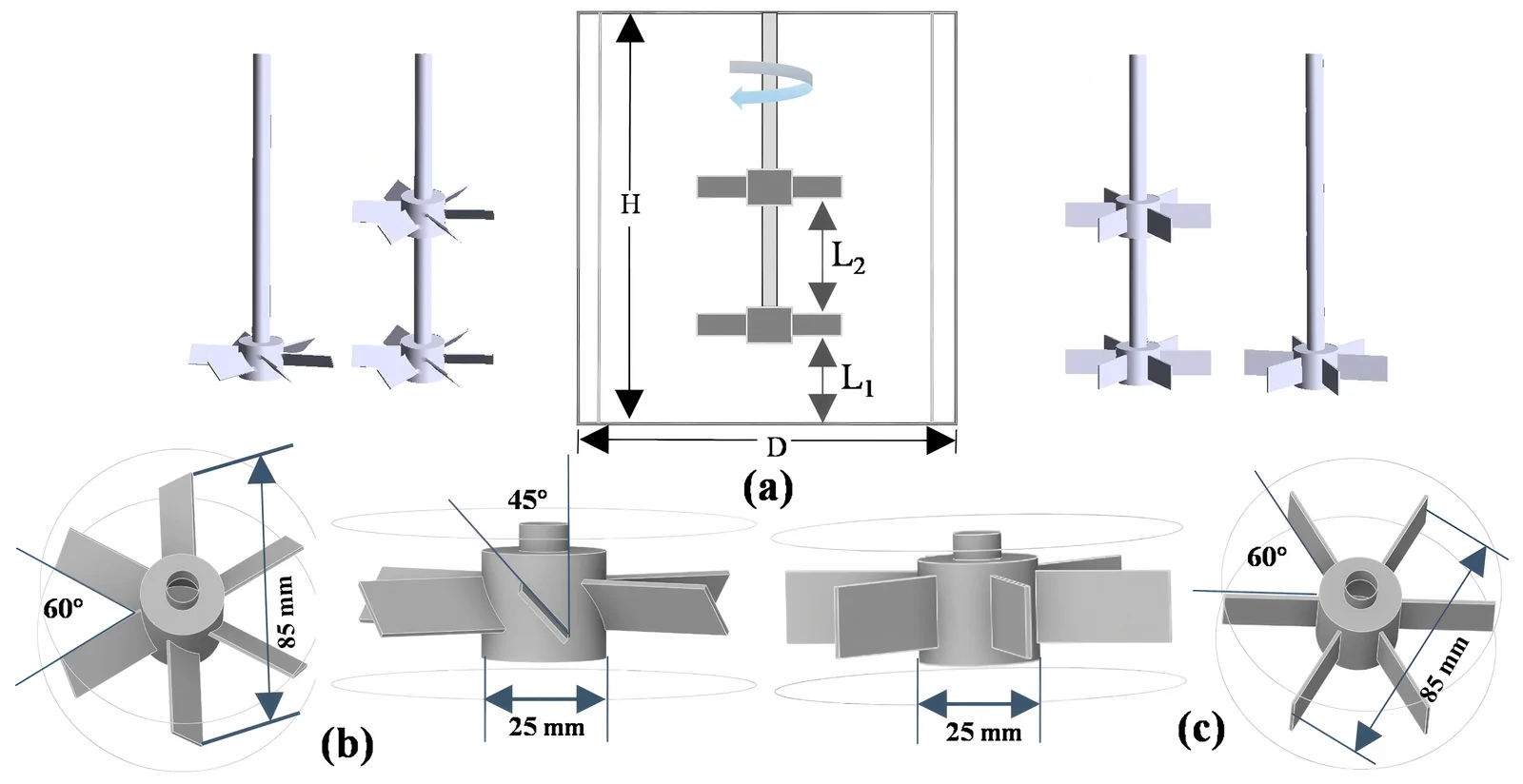
(**a**) Reactor geometry, (**b**,**c**) impeller geometries.

**Figure 2:**
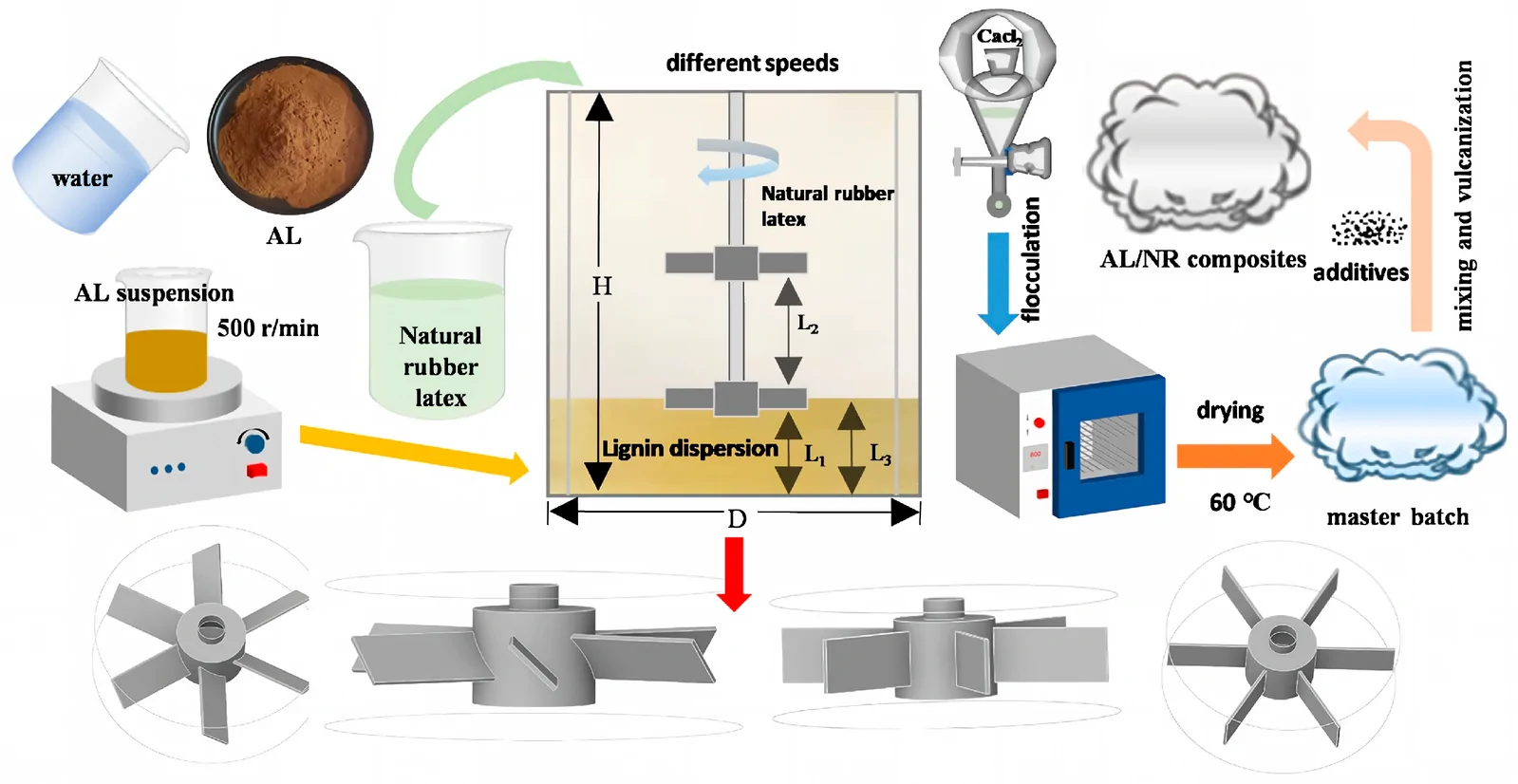
Schematic illustration of the preparation of AL/NR composites.

**Figure 3:**
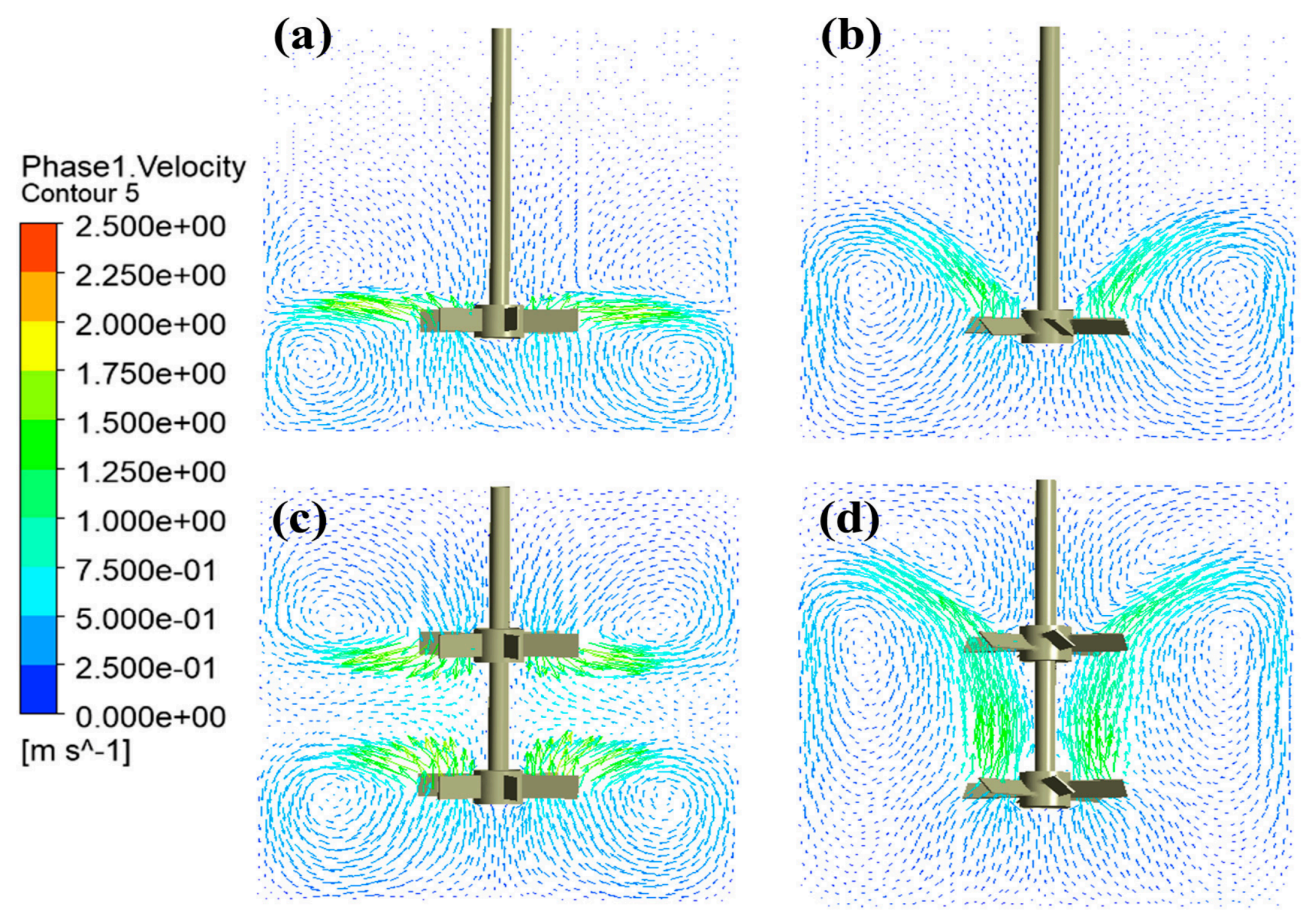
Velocity vector distributions on the central longitudinal section at 600 rpm: (**a**) SSB, (**b**) S45P, (**c**) DSB, (**d**) D45P.

**Figure 4:**
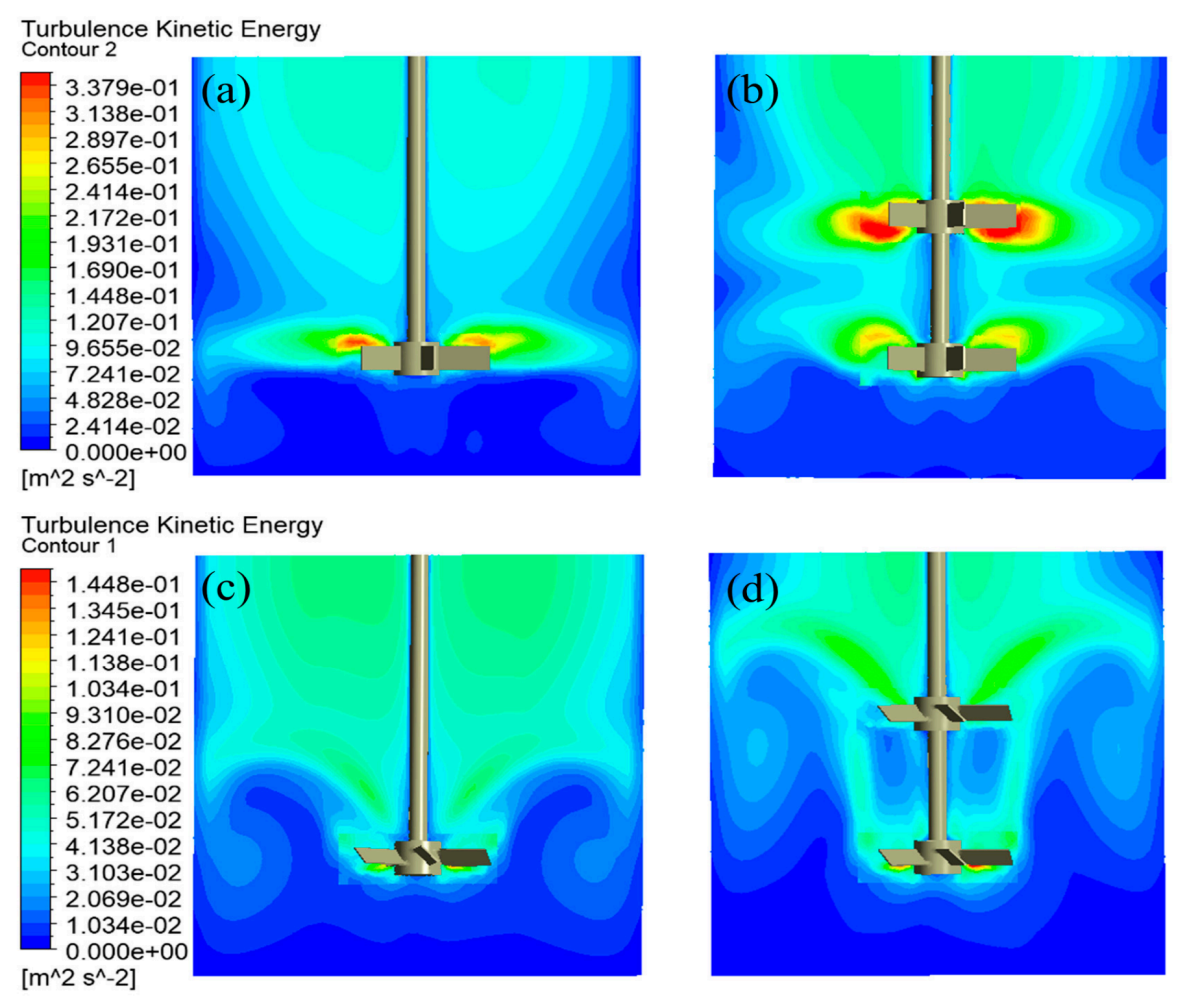
Contours of turbulent kinetic energy on the central longitudinal section at 600 rpm: (**a**) SSB, (**b**) DSB, (**c**) S45P, (**d**) D45P.

**Figure 5:**
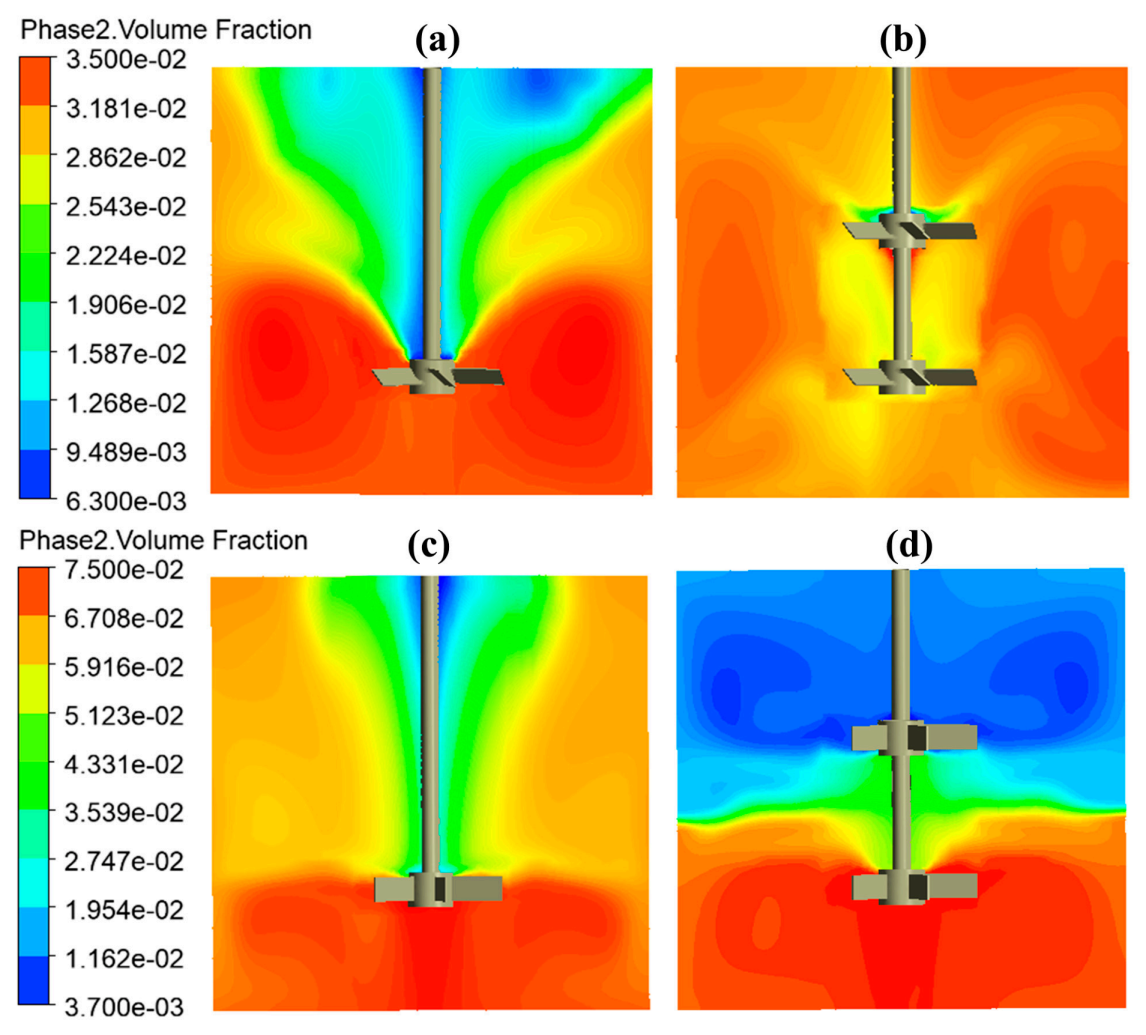
AL volume fraction contours on the central longitudinal section at 600 rpm: (**a**) S45P, (**b**) D45P, (**c**) SSB, (**d**) DSB.

**Figure 6:**
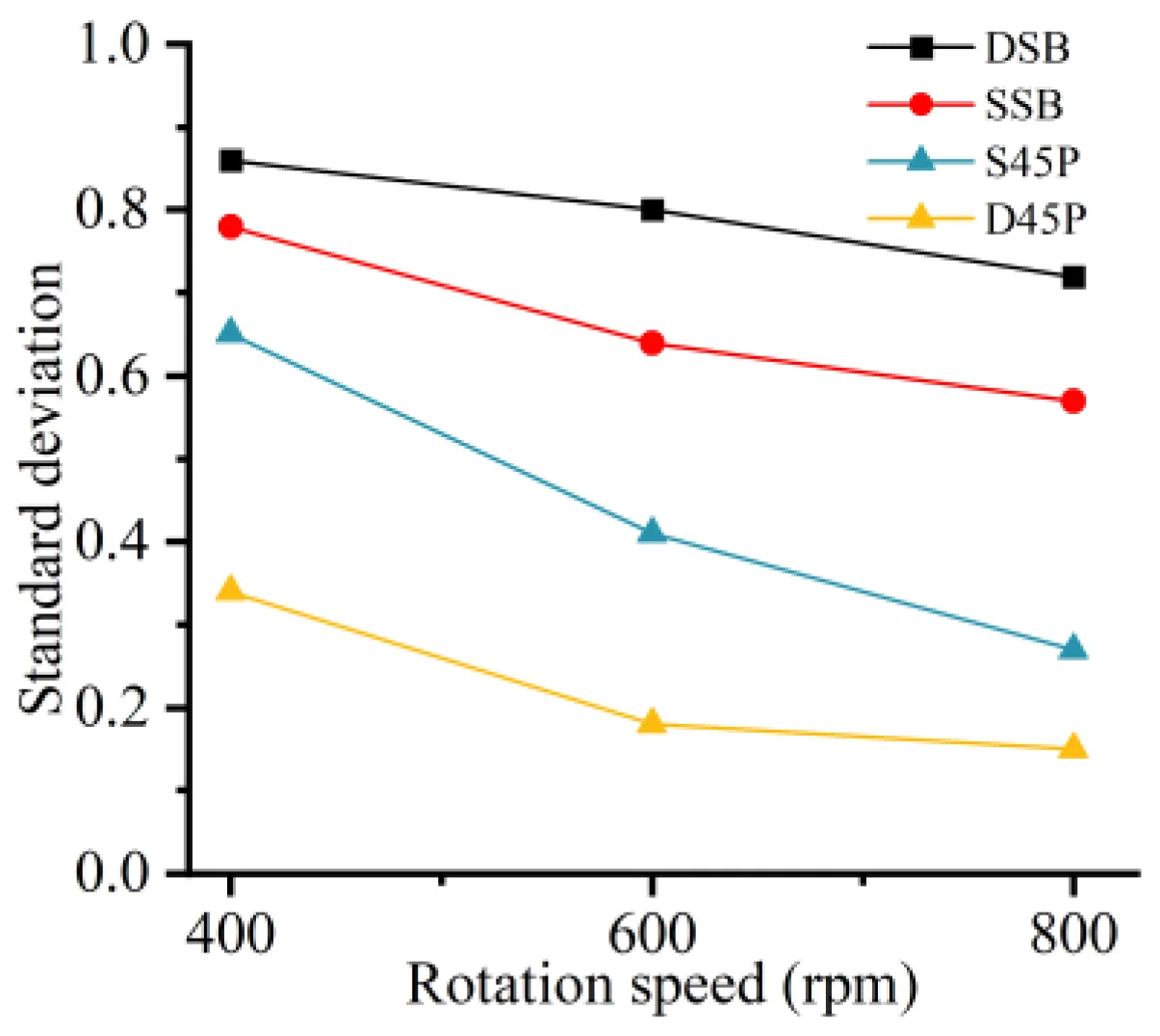
Weighted volume fraction standard deviation of the AL phase at different agitation speeds.

**Figure 7:**
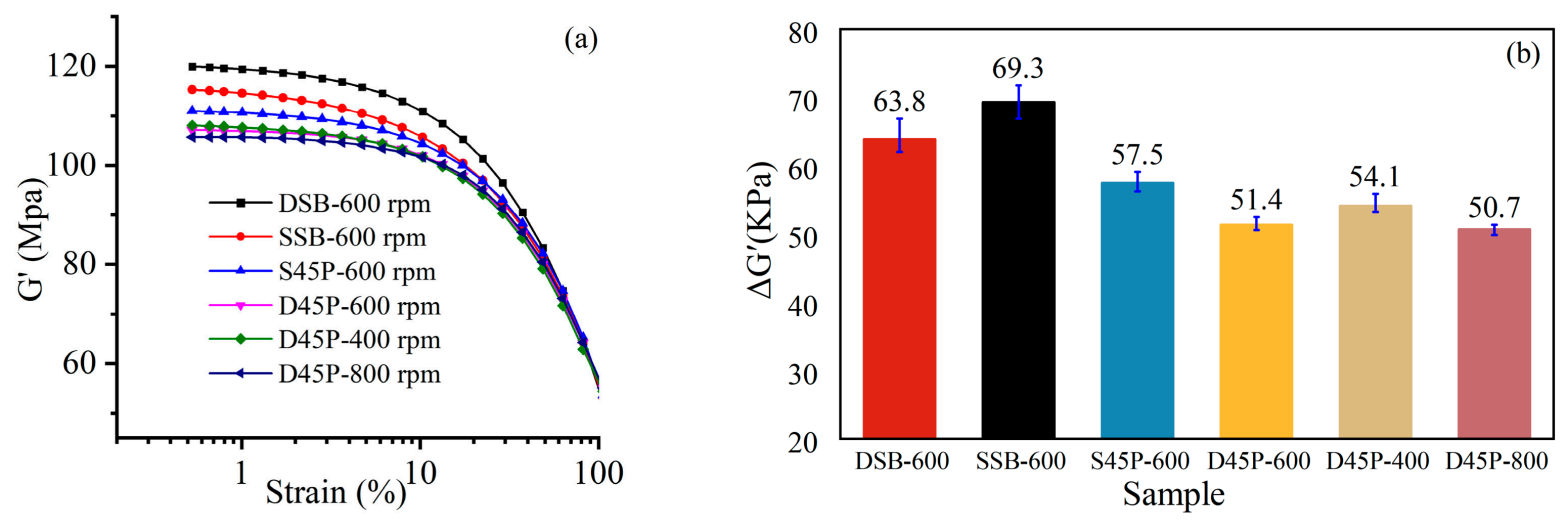
RPA test results including (**a**) G′ vs strain curves and (**b**) ΔG′.

**Figure 8:**
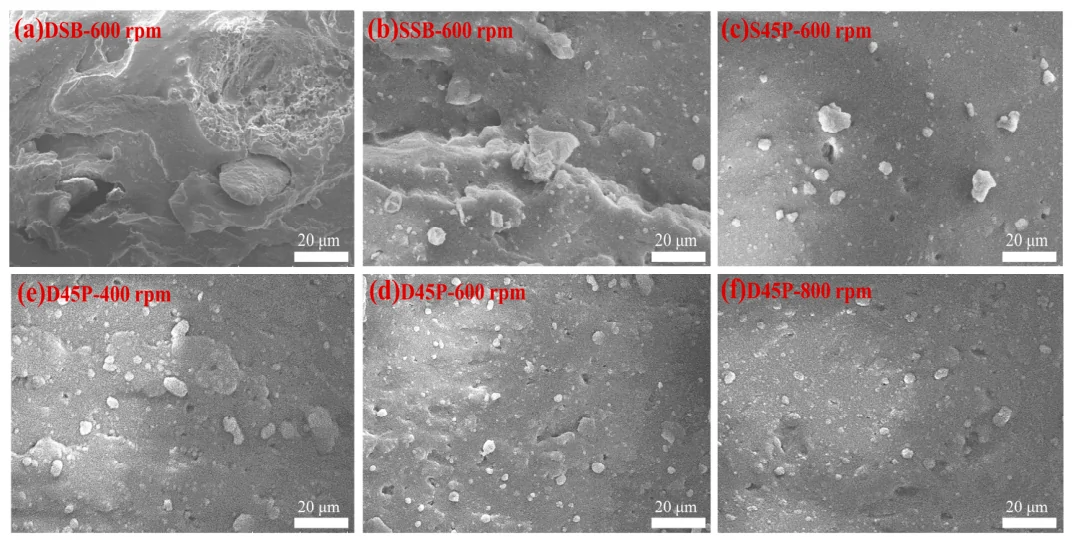
SEM images of tensile fractured surfaces of AL/NR composites: (**a**) DSB, 600 rpm; (**b**) SSB, 600 rpm; (**c**) S45P, 600 rpm; (**d**) D45P, 600 rpm; (**e**) D45P, 400 rpm; (**f**) D45P, 800 rpm.

**Figure 9:**
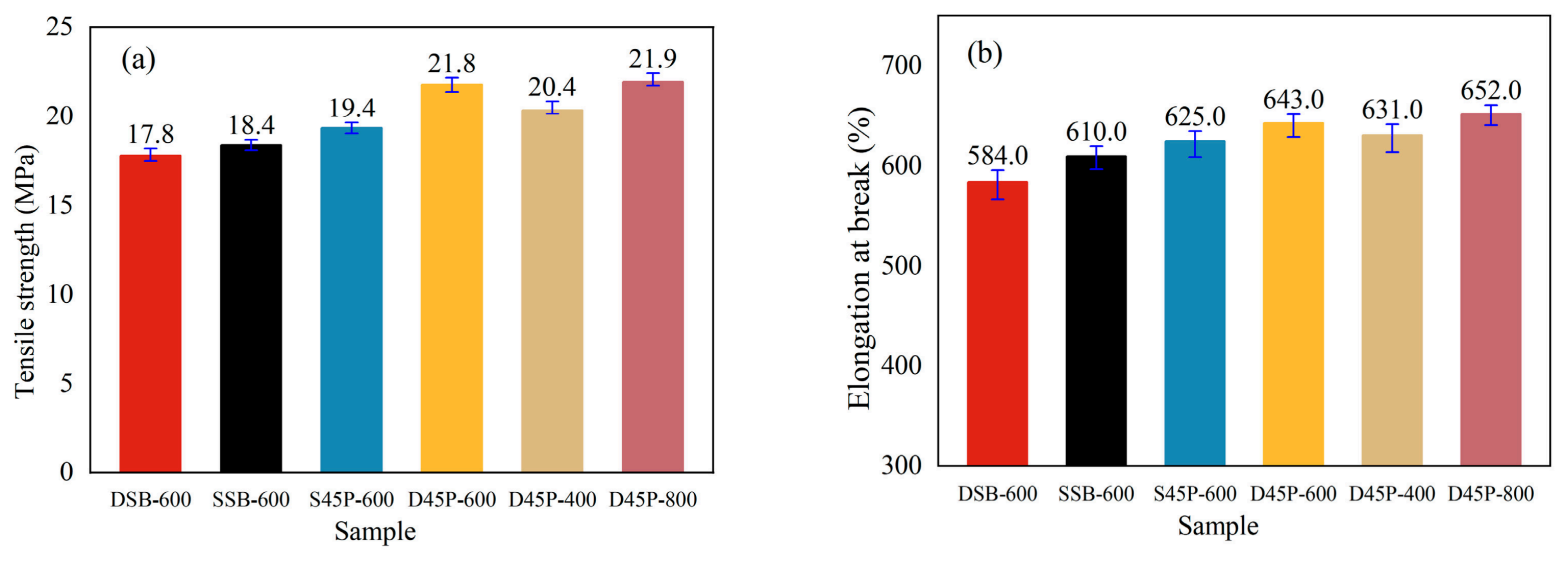
Physical and mechanical properties of AL/NR composites: (**a**) tensile strength and (**b**) elongation at break.

**Figure 10:**
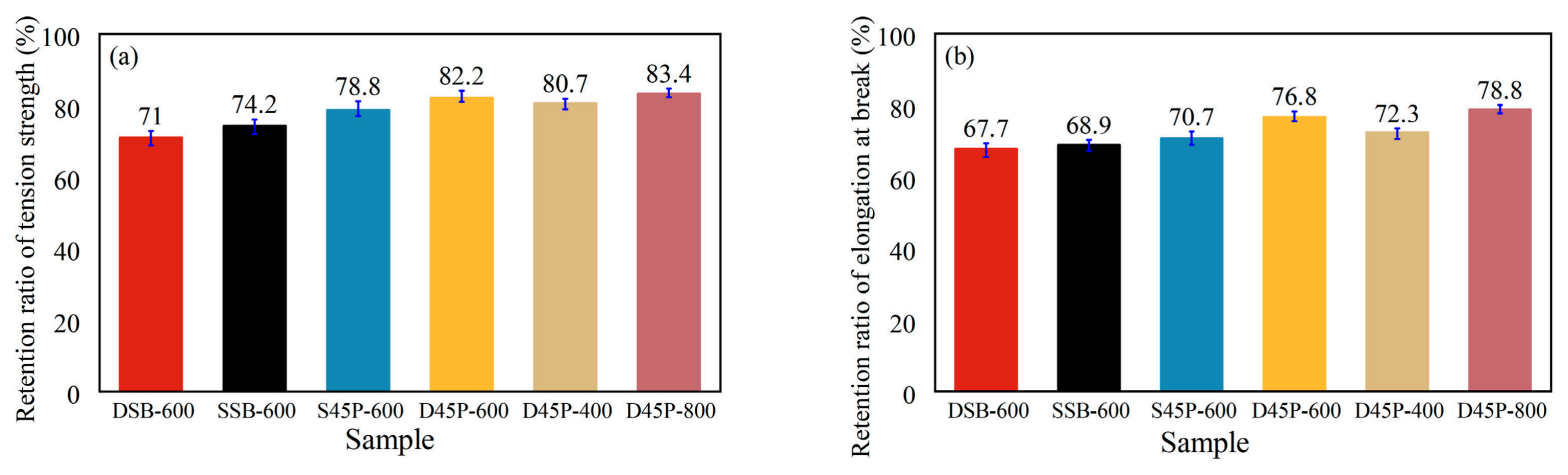
Thermo-oxidative aging properties of the AL/NR composites: (**a**) retention rate of tensile strength and (**b**) retention rate of elongation at break.

**Table 1:** Grid independence verification results at 800 rpm.

Mesh Number	D45P-800	S45P-800	DSB-800	SSB-800
250,000	0.27	0.36	0.83	0.69
680,000	0.16	0.27	0.72	0.57
960,000	0.15	0.26	0.69	0.55

**Table 2:** Vulcanization properties of the AL/NR composites.

Sample	MH (dN·m)	ML (dN·m)	MH−ML (dN·m)	t_10_ (min)	t_90_ (min)
DSB-600	14.19	1.89	12.30	2.65	15.78
SSB-600	15.43	1.77	13.66	2.87	16.29
S45P-600	15.76	1.69	14.07	3.09	16.80
D45P-600	15.93	1.62	14.31	3.28	17.22
D45P-400	15.84	1.67	14.17	3.12	16.88
D45P-800	15.99	1.53	14.46	3.34	17.35

## Data Availability

The original contributions presented in this study are included in the article. Further inquiries can be directed to the corresponding authors.

## Abbreviations

The following abbreviations are used in this manuscript:

AL	Alkali lignin
NR	Natural rubber
NRL	Natural rubber latex
ENR	Epoxidized natural rubber
LNP	Lignin nanoparticle
CFD	Computational fluid dynamics
MRF	Multiple reference frame
TKE	Turbulent kinetic energy
RPA	Rubber processing analyzer
SEM	Scanning electron microscopy
ML	Minimum torque
MH	Maximum torque
t10	Scorch time
t90	Optimum cure time
SSB	Single-stage straight-blade impeller
DSB	Two-stage straight-blade impeller
S45P	Single-stage 45° pitched-blade impeller
D45P	Two-stage 45° pitched-blade impeller
